# Type of cycle, temperament and childhood trauma are associated with lithium response in patients with bipolar disorders

**DOI:** 10.1186/s40345-024-00331-0

**Published:** 2024-04-02

**Authors:** Delfina Janiri, Alessio Simonetti, Mario Luciano, Silvia Montanari, Evelina Bernardi, Giuseppe Carrà, Andrea Fiorillo, Gabriele Sani

**Affiliations:** 1 Department of Psychiatry, Fondazione Policlinico Universitario Agostino Gemelli IRCCS, Rome, Italy; 2 Department of Neurosciences, Section of Psychiatry, Università Cattolica del Sacro Cuore, Rome, Italy; 3 Menninger Department of Psychiatry and Behavioral Sciences, Baylor College of Medicine, Houston, TX USA; 4 Department of Psychiatry, University of Campania “L. Vanvitelli”, Naples, Italy; 5 Department of Medicine and Surgery, University of Milano-Bicocca, Monza, Italy; 6 Division of Psychiatry, University College London, London, UK

**Keywords:** Lithium response, Temperament, Childhood trauma, Type of cycle

## Abstract

**Background:**

Lithium stands as the gold standard in treating bipolar disorders (BD). Despite numerous clinical factors being associated with a favorable response to lithium, comprehensive studies examining the collective influence of clinical variables alongside psychopathological dimensions are lacking. Our study aims to enhance comprehension of lithium response in individuals with BD by integrating clinical variables with psychopathological traits and early adverse events.

**Methods:**

We assessed 201 patients with BD for clinical characteristics, childhood trauma, temperament traits, impulsivity, and aggression. Lithium response was evaluated using the gold standard Alda scale, and predictors of lithium response were estimated through a multivariate model.

**Results:**

On the total sample, 61 (30.3%) patients were lithium responders according to the Alda scale. Comparatively, lithium responders, in contrast to non-responders, demonstrated a higher prevalence of the mania-depression-interval (MDI) cycle, a more frequent diagnosis of BD type I, and reported an earlier age of onset. They also exhibited less lifetime substance abuse, emotional, physical, and sexual abuse, while scoring higher on hyperthymic and irritable temperament scales. In multivariate analyses, only the MDI cycle (OR,3.47; 95%CI,1.61–7.50) hyperthymic (OR,1.20; 95%CI,1.02–1.41) and irritable temperament (OR,1.28; 95%CI,1.08–1.52) persisted as significant predictors of a positive response to lithium treatment, while emotional (OR,0.87; 95%CI,0.76–0.98) and physical abuse (OR,0.83; 95%CI,0.70–0.98) were predictors of non-response.

**Conclusions:**

In evaluating lithium response in BD, our study highlights the importance of considering clinical variables alongside temperament and childhood adversities. The assessment of hyperthymic and irritable temperament, emotional and physical abuse together with the type of cycle is of particular importance. Furthermore, our findings underscore the significance of systematically assessing the type of cycle in patients with BD through the use of life charts.

## Background

Bipolar disorder (BD) is a chronic mental health condition subjecting individuals to a relentless cycle of mood episodes (Goodwin and Jamison 2007). With an 80% risk of episode recurrence and a substantial prevalence of comorbid mental and physical health conditions, BD poses a substantial burden on individuals and society at large, with an elevated risk of all-cause mortality and suicide (Hayes et al. 2015). Untimely and insufficient treatments further exacerbate the challenges experienced by individuals with BD, compounding the burden of the disorder.

Amidst this complex landscape, lithium has emerged as a cornerstone in the long-term therapy of BD. In particular, previous research has shown that lithium monotherapy is especially effective in a subset of patients, as it has the capacity to greatly influence their lives by preventing recurring mood episodes (Grof 1999). A significant proportion of patients on lithium treatment, about 25–30%, fall into the category of “excellent lithium responders” (Garnham et al. 2007; Grof 2010; Rybakowski et al. 2001). These individuals frequently display distinct clinical characteristics (Grof 2010).

A recent systematic review and meta-analysis (Hui et al. 2019) have discerned six clinical predictors that distinctly portend a favorable response to lithium: the presence of a mania-depression-interval sequence, the absence of rapid cycling, the absence of psychotic symptoms, a family history of BD, a shorter pre-lithium illness duration, and a later age of onset. However, the authors of this meta-analysis cautioned that the existing body of literature on clinical predictors of lithium response warrants careful interpretation, particularly given the variations in the definition and measurement of lithium response across the included studies (Hui et al. 2019). A potential means to address this challenge lies in the systematic application of the ‘Retrospective Criteria of Long-Term Treatment Response in Research Subjects with Bipolar Disorder,’ commonly known as the Alda scale (Grof et al. 2002). The Alda scale has been employed in multiple studies and has demonstrated substantial inter-rater agreement and reliability (Manchia et al. 2013). Recent studies leveraging the Alda scale in a sizable sample of patients with BD have confirmed that lithium responsiveness can be predictably assessed using clinical markers (Nunes et al. 2020; Scott et al. 2020). However, they underscore the need for refinement in classification, recognizing the considerable heterogeneity in the clinical profiles of patients with BD.

To address this challenge, one potential avenue is to expand the assessment beyond the clinical course and incorporate specific psychopathological characteristics of BD individuals. Within the realm of psychopathological traits, particular attention has been given to impulsivity and aggressive tendencies, as these factors are linked to BD and may delineate varying patients’ clinical profiles (Janiri et al. 2018).

Notably, no study to date has explored the relationship between impulsivity/ aggression and lithium response, while two prior investigations searched the interplay between temperament traits and early adversities with treatment response (Etain et al. 2016; Rybakowski et al. 2013). Affective temperaments, as conceptualized by Hagop Akiskal (Akiskal 2000), represent enduring and stable patterns of emotional reactivity and regulation that influence an individual’s overall mood disposition. Only one study has investigated temperament traits in relation to lithium response in BD (Rybakowski et al. 2013); using the Alda scale and the Temperament Scale of Memphis, Pisa, Paris, and San Diego-Autoquestionnaire short form (TEMPS-A-SF), researchers uncovered a significant positive correlation between lithium response and hyperthymic temperament scores.

In parallel, early adverse events have been implicated in the heterogeneity of the clinical profiles of psychiatric patients (Daruy-Filho et al. 2011; Janiri et al. 2018). Specifically, previous observations highlighted childhood traumatic events as predictors of nonresponse to psychotropic drugs in psychiatric treatment (Douglas and Porter 2012). Nevertheless, a recent meta-analysis warned about the small body of research identified on the effect of childhood trauma on the effectiveness of specific pharmacological/psychological interventions (Wrobel et al. 2022). One specific study, sought to determine whether childhood trauma predisposes individuals to inadequate responses to lithium using the Alda scale (Etain et al. 2016). This study revealed that early adverse events, particularly physical abuse, were associated with poor responses to lithium.

The inquiry into whether temperament and childhood trauma independently or in conjunction with other psychopathological dimensions like aggression and impulsivity, along with distinct clinical presentations, influence response to lithium treatment remains an inadequately explored line of research. Therefore, the primary objective of the present study is to address this gap in knowledge by examining the relationship between lithium response, assessed through the Alda scale, temperament traits, childhood adversities, and a comprehensive range of clinical and psychopathological factors. This investigation will encompass a substantial sample of patients diagnosed with bipolar disorder, both type I and type II.

## Methods

### Participants

Outpatients with DSM-5 diagnoses of BD type I and BD type II were recruited at the Psychiatry Department of the Fondazione Policlinico Universitario Agostino Gemelli IRCCS in Rome, Italy, starting from June 2022. Diagnosis was confirmed using the Structured Clinical Interview for DSM-5 (First 2015). Diagnostic interviews were conducted by two trained assessors with demonstrated high interrater reliability (k = 0.87). By implementing stringent inclusion and exclusion criteria, the study aimed to ensure a representative and reliable sample for the investigation. In addition to a DSM-5 diagnosis of BD, inclusion criteria were as follows: (a) age 18‐65 years; (b) at least 5 years of education; (c) fluency in Italian; (d) stable pharmacological treatment for at least 6 months. Exclusion criteria were: (a) a history of psychosis unrelated to the primary mood disorder; (b) traumatic brain injury with loss of consciousness; (c) major medical or neurological conditions; (d) a Mini‐Mental State Examination (MMSE) score of less than 24 (since scores below this level indicate cognitive deterioration based on normative data from the Italian population); (e) current substance use disorder. Patients were in clinical remission according to the Young Mania Rating Scale (YMRS) for manic symptoms and the 17-item Hamilton Rating Scale for Depression (HAMD) for depressive symptoms.

Based on the above inclusion/exclusion criteria, we consecutively enrolled 201 patients in this study.

The study conformed to the Principles of Human Rights, as adopted by the World Medical Association at the 18th WMA General Assembly in Helsinki, Finland, in June 1964 and subsequently amended at the 64th WMA General Assembly in Fortaleza, Brazil, in October 2013. All participants gave written informed consent to participate in the study after receiving a full explanation of the study procedures and objectives. Patients received no financial compensation for this study. The commitment to ethical standards, as reflected in adherence to the World Medical Association’s principles, underscored the importance of safeguarding participant rights and well-being throughout the research process. The thoroughness of diagnostic procedures and ethical safeguards contributes to the robustness and reliability of the study’s findings, enhancing the scientific and ethical integrity of the research endeavor. The study was approved by local ethics committees (protocol number: 5016).

#### Clinical assessment

A semi-structured interview, employed in prior studies (Janiri et al. 2019), was utilized for comprehensive data collection on anamnestic characteristics and clinical information. Administered by an experienced psychiatrist, this interview adhered to DSM-5 criteria and clinical assessments, steering clear of simplistic yes/no responses to ensure nuanced insights. Question wording was adaptable for clarity, and the final evaluation incorporated inputs not only from the patients but also from family members/close friends (who were consistently present for at least one visit) and relevant medical records. All gathered data, spanning family history, psychiatric background, and current psychiatric status, were meticulously entered into preprinted medical records. Course sequences were evaluated based on life charts previously employed in other studies (Koukopoulos et al. 2013). This assessment facilitated the identification of both the sequences and severity of episodes. It also allowed for the characterization of major episode sequences concerning the order of pairs of major or minor depressive and manic, or hypomanic episodes, as well as euthymic intervals.

#### Lithium response assessment

To evaluate the response to lithium treatment, we utilized the Alda scale, specifically designed for the retrospective assessment of prophylactic treatment responses in naturalistic settings. This scale consists of two distinct subscales, labeled as A and B.

The A scale, with a scoring range of 0 to 10, assesses the extent of improvement attributed to lithium treatment, such as reductions in recurrence frequency and residual symptoms, among other factors. The B scale comprises five criteria, each rated from 0 to 2, with higher scores indicating a greater potential for external factors undermining the observed improvement attributed to lithium treatment. These five potential confounding factors incorporated in the B scale are as follows: (B1) Number of episodes before / off the treatment, (B2) Frequency of episodes before / off the treatment, (B3) Duration of lithium treatment, (B4) Compliance during period(s) of stability, and (B5) Use of additional medication during the period of stability. The Total Score (TS) on the Alda scale is calculated by subtracting the B score from the A score. If the B scale score exceeds the A scale score, resulting in a negative score, it is recorded as 0. In line with Manchia et al. (Manchia et al. 2013), a TS value equal to or greater than 7 was considered indicative of a respondent phenotype. It is important to note that while the Alda Scale was originally designed for evaluating long-term treatment responses, we chose to include participants with less than one year of lithium treatment in our study. This decision was made because the scale accounts by potential bias due to the duration of the treatment under Criteria B3. It is worth mentioning that only a small percentage (2.5%) of our sample had been receiving lithium treatment for less than one year.

#### Temperament traits and psychopathological assessment

We assessed temperaments, including cyclothymic, dysthymic, irritable, hyperthymic, and anxious temperaments, using the short form of the TEMPS-A. The questionnaire items were derived from the validated Italian version of this instrument (Preti et al. 2010).

To evaluate aggressive tendencies, we employed the Aggression Questionnaire (AQ) (Buss and Perry 1992), a self-report questionnaire which consists of 29 items and identifies four dimensions of aggression: physical aggression, verbal aggression, anger, and hostility. Responses to all items were recorded on a five-point Likert scale, ranging from ‘never’ (1) to ‘always’ (5). Subscale scores were summated to yield an overall score, with higher scores indicating greater aggression.

Impulsiveness was assessed using the Barratt Impulsiveness Scale (BIS), a 30-item self-report inventory. The scale provides a total score measuring overall impulsiveness and three second-order factors, including attentional (lack of focus on a task), motor (quick reactions, restlessness), and non-planning (preference for the present over the future) impulsiveness. Participants rated items on a scale from 1 (rarely) to 4 (almost always/always), with eleven of the items being reverse-scored. The total score ranges from 0 to 120, with higher scores indicating greater trait impulsiveness.

To assess adverse childhood events, we employed the Italian short form of the Childhood Trauma Questionnaire (CTQ-SF) (Bernstein et al. 2003), a 28-item retrospective self-report questionnaire. This instrument investigates traumatic experiences during childhood and rates each item on a 5-point Likert scale, ranging from 1 = “never true” to 5 = “very often true,” based on the frequency of the events. The questionnaire assesses five types of trauma: emotional abuse, emotional neglect, physical abuse, physical neglect, and sexual abuse. Scores are calculated for both the total scale (range 25–125) and each type of trauma (range 5–25).

### Statistical analyses

To fit our aim, we subdivided our sample into two groups: patients lithium responders and non-responders, according to the Alda scale cut-off (TS ≥7 = good lithium responders).

Analyses used standard univariate/bivariate comparisons of continuous measures (ANOVA) and categorical measures (contingency table/ χ2) to compare factors of interest (including sociodemographic, clinical, psychopathological characteristics, and temperament traits) in the two groups. The level of significance was set at *p* < 0.05.

Furthermore, for the first aim of this study, we decided to test the associations of the variables of interest with lithium response using a multivariate model. Specifically, the model focused on the predictive value of the variables of interest on the outcome. All factors significantly associated with lithium response (all the variables significantly different in the bivariate analyses), along with sex/gender and age as covariates, were subjected to a binary logistic regression to generate Odds Ratios (ORs) and their 95% confidence intervals (CIs), with lithium response as dependent outcome measures. We examined possible multicollinearity between variables of interest using variance inflation factor (VIF) indicator obtained from a linear regression analysis. Regarding psychopathological characteristics, significance regarded the scores of the subscales rather than those of the total scales. We used the statistical routines of SPSS Statistics 26.0 for Windows (IBM Co., Armonk, New York, USA).

## Results

On the total sample, 61 (30.3%) patients were lithium responders according to the Alda scale. With regard to the Alda Scale ratings, the sample mean TS was 4.26 (SD: 2.50), with the mean A scale score being 6.95 (SD: 1.77) and the mean B scale score being 2.69 (SD:1.63). In the total sample mean age was 44.36 (SD:12.52) and mean education was 14.26 (SD:3.50). In total 103 individuals were males (51.2%).

Sociodemographic and clinical characteristics of the sample are shown in Table 1. The two groups of patients did not differ for sociodemographic and life-style characteristics. With respect to clinical characteristics, univariate/bivariate analyses showed that the two groups differed for diagnosis, substance use during life- time, age at onset and type of cycle (Table 1). In particular, lithium responders were more frequently diagnosed as BD type I, reported less lifetime substance abuse, and an early age at onset (Table 1). With regard to the type of cycle, post-hoc analyses specified that lithium responders, compared to lithium non-responders, presented more frequently with mania-depression-interval (MDI) cycle than both depression-mania-interval (DMI) (X^2^ = 11.80, df = 1, *p* = 0.001) and irregular cycle (X^2^ = 4.21, df = 1, *p* = 0.04). The two groups did not differ between DMI cycle and irregular (X^2^ = 0.20, df = 1, *p* = 0.65).

**Fig. 1:**
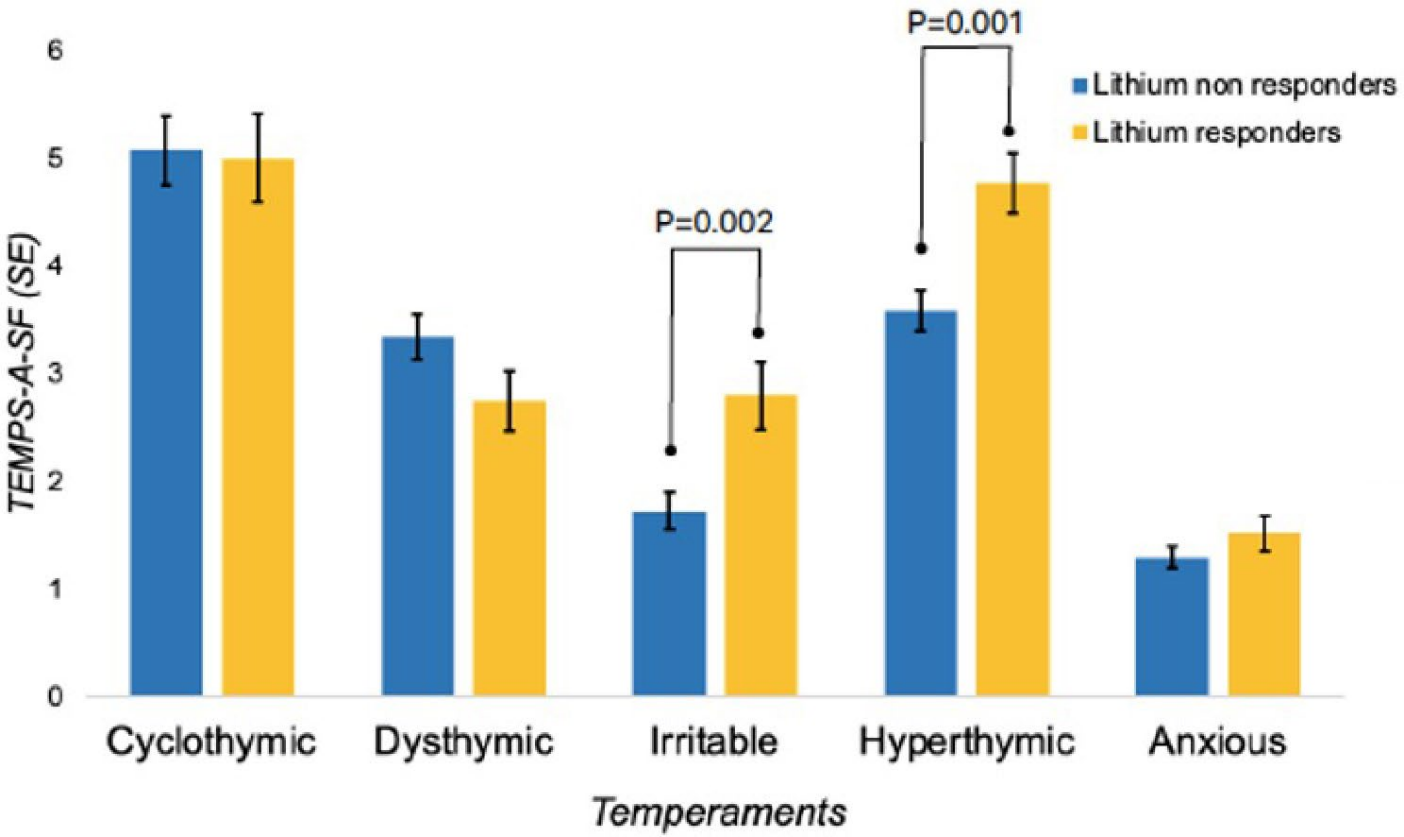
Distribution patterns of temperaments trait in lithium responders and non-responders

**Fig. 2:**
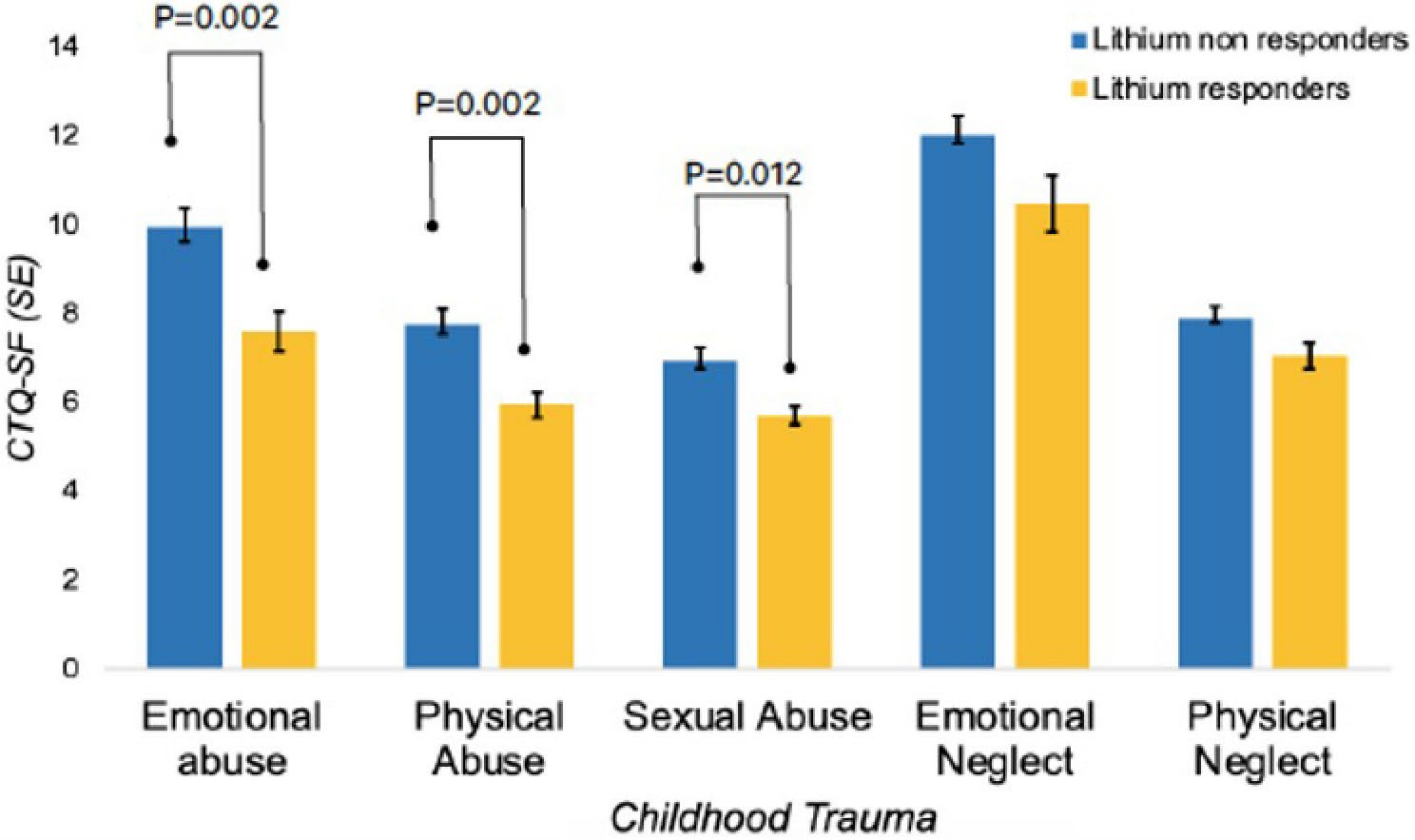
Distribution patterns of childhood traumatic experiences in lithium responders and non-responders

In terms of temperament traits and psychopathological characteristics, lithium responders scored higher in the hyperthymic and irritable temperament than non-responders (Table 2; Fig. 1). They also reported less childhood emotional, physical, and sexual abuse than non-responders (Table 2; Fig. 2).

Multivariate logistic regression model (*p* < 0.001) revealed that hyperthymic and irritable temperament, together with MDI cycle, were specific predictors of lithium response, whereas childhood emotional and physical abuse were predictors of non-response (Table 3). There was no significance of multicollinearity, as indicated by the fact that VIF of all variables of interest was < 2.

**Table 1:** Sociodemographic and clinical characteristics of the sample according to lithium response (*N* = 201)

	Lithium responders (*n* = 61)	Lithium non-responders (*n* = 140)	F or χ2	df	P
*Socio-demographic and lifestyle characteristics*					
Age, y - mean ± SD	43.48 ± 13.15	44.75 ± 12.27	0.432	1	0.512
Gender - n(%)			0.15	1	0.699
Males	30 (49.2)	73 (52.1)			
Females	31 (50.8)	67 (47.9)			
Education, y - mean ± SD	14.85 ± 3.39	14.01 ± 3.53	2.49	1	0.116
Married partner - n(%)	26 (42.6)	67 (47.9)	0.47	1	0.494
Coffee, yes – n(%)	48 (78.7)	111 (79.3)	0.01	1	0.924
Smoking, yes - n(%)	29 (47.5)	68 (48.6)	0.02	1	0.893
Substance use, lifetime - n(%)	8 (13.1)	40 (28.6)	5.58	1	0.018*
BMI - mean ± SD	25.87 ± 4.22	25.73 ± 3.73	0.06	1	0.813
*Clinical variables*					
Diagnostic status - n(%)			4.74	1	0.030*
BD I	53 (86.9)	102 (72.9)			
BD II	8 (13.1)	38 (27.1)			
Type of cycle			13.15	2	0.001**
MDI	39 (63.9)	51 (36.4)			
DMI	15 (24.6)	65 (46.4)			
IRR	7 (11.5)	24 (17.1)			
Age at onset, y - mean ± SD	25.65 ± 9.67	29.18 ± 10.85	4.78	1	0.030*
Family history of psychiatric disorders - n(%)	42 (68.9)	95 (67.9)	0.019	1	0.889
Seasonality - n(%)	23 (37.7)	53 (37.9)	0.00	1	0.984
Switch - n(%)	24 (39.3)	45 (32.1)	0.98	1	0.323
Psychotic symptoms – n(%)	38 (62.3)	68 (48.6)	3.21	1	0.073
Hospitalizations - n(%)	37 (60.7)	102 (72.9)	2.96	1	0.085
Suicidal ideation - n(%)	39 (63.9)	82 (58.6)	0.51	1	0.475
Suicidal attempts - n(%)	21 (35.6)	38 (27.1)	1.09	1	0.297

*p < 0.05; **p < 0.01; ***p < 0.001; df, degrees of freedom; P, significance level; SD, standard deviation; n, number of observations; y, years; BMI, Body Mass Index; BD I, Bipolar Disorder type I; BD II, Bipolar Disorder type II; MDI, Mania-Depression-Interval; DMI, Depression-Mania-Interval; IRR, Irregular

**Table 2:** Psychopathological dimensions, temperament traits and childhood trauma according to lithium response (*n* = 201)

	Lithium responders (*n* = 61)	Lithium non-responders (*n* = 140)	F	df	P
*TEMPS-A-SF*					
Cyclothymic	5.08 ± 3.78	5.07 ± 3.20	0.001	1	0.981
Dysthymic	3.35 ± 2.48	2.75 ± 2.16	2.63	1	0.106
Irritable	1.72 ± 1.96	2.80 ± 2.50	10.33	1	0.002**
Hyperthymic	3.59 ± 2.32	4.78 ± 2.17	11.39	1	0.001**
Anxious	1.30 ± 1.21	1.52 ± 1.31	1.27	1	0.260
*CTQ-SF*					
Emotional abuse	9.94 ± 5.30	7.61 ± 3.47	9.87	1	0.002**
Physical Abuse	7.74 ± 4.27	5.96 ± 2.19	9.49	1	0.002**
Sexual Abuse	6.94 ± 3.64	5.70 ± 1.50	6.49	1	0.012*
Emotional Neglect	12.03 ± 5.18	10.49 ± 4.98	3.78	1	0.053
Physical Neglect	7.89 ± 3.08	7.06 ± 2.41	3.45	1	0.065
*BIS*					
Attentional	15.84 ± 3.93	15.18 ± 3.58	1.10	1	0.295
Motor	22.09 ± 4.71	21.35 ± 4.12	1.01	1	0.316
Non-planning	26.81 ± 5.05	25.80 ± 5.46	1.45	1	0.230
Total	64.80 ± 10.66	62.33 ± 10.36	2.08	1	0.151
*AQ*					
Physical	17.12 ± 7.04	16.58 ± 5.84	0.235	1	0.628
Verbal	14.70 ± 4.34	13.85 ± 3.49	1.64	1	0.202
Hanger	16.78 ± 5.52	16.87 ± 4.96	0.01	1	0.920
Hostility	20.56 ± 6.74	19.59 ± 5.37	0.89	1	0.347
Total	69.05 ± 18.63	67.28 ± 15.23	0.384	1	0.536

*p < 0.05; **p < 0.01; ***p < 0.001; df, degrees of freedom; P, significance level; SD, standard deviation; TEMPS-A-SF, Temperament Evaluation of Memphis, Pisa, Paris and San Diego – Autoquestionnaire, short form; CTQ-SF, Childhood Trauma Questionnaire short form; BIS, Barratt Impulsiveness Scale; AQ, Aggression Questionnaire

**Table 3:** Predictors of lithium response according to multivariate logistic regression

	OR	95% CI	Wald	P
Lithium responders vs. Non-responders				
Age	1.01	0.97–1.04	0.15	0.695
Gender	0.52	0.23–1.17	2.48	0.115
Diagnosis	2.35	0.90–6.15	3.01	0.083
Age at onset	0.96	0.91-1.00	3.36	0.067
Substance use	2.53	0.93–6.88	2.28	0.070
MDI cycle	3.47	1.61–7.50	10.01	0.002**
TEMPS-A-SF Hyperthymic	1.20	1.02–1.41	4.66	0.031*
TEMPS-A-SF Irritable	1.28	1.08–1.52	8.18	0.004**
CTQ-SF Emotional Abuse	0.87	0.76–0.98	4.95	0.026*
CTQ-SF Physical Abuse	0.83	0.70–0.98	4.57	0.033*
CTQ-SF Sexual Abuse	0.98	0.79–1.21	0.04	0.850

*p < 0.05; **p < 0.01; ***p < 0.001; df, degrees of freedom; P, significance level; SD, standard deviation; MDI, Mania-Depression-Interval; TEMPS-A-SF, Temperament Evaluation of Memphis, Pisa, Paris and San Diego–Auto-questionnaire, short form; CTQ-SF, Childhood Trauma Questionnaire short form

## Discussion

In the present study, we conducted an investigation in a cohort of patients diagnosed with both BD type I and type II to explore the connection between lithium response and a comprehensive set of variables, encompassing not only clinical factors but also temperament traits and psychopathological dimensions. Our findings revealed that among clinical factors, only the presence of a manic-depressive-interval (MDI) cycle emerged as a specific predictor of lithium response. In terms of temperament traits, both hyperthymic and irritable temperaments were identified as predictors of positive lithium response, while a history of childhood emotional and physical abuse was indicative of non-response to lithium treatment.

The observation that the MDI cycle type can be a clinical predictor of lithium response aligns with existing literature. Clinical studies conducted in the 1980s observed a higher occurrence of the MDI sequence in patients who displayed a favorable response to lithium compared to those with poor responses (Grof et al. 1987; Haag et al. 1987; Koukopoulos et al. 2013; Maj et al. 1989). Authors postulated that this phenomenon might be associated with the primacy of antimanic action of lithium during manic/hypomanic phases, as it may serve as a protective measure against subsequent episodes of depression in MDI cycles (Koukopoulos et al. 2013; Maj et al. 1989). Authors also highlighted that the course characterized by depression-mania-interval (DMI) is frequently associated with antidepressant treatment for the initial depression, often resulting in mood switches into mania or hypomania (Maj et al. 1989). Such mood switch tended to be relatively resistant to the effects of mood stabilizers.

It is worth noting that in these earlier studies the response to lithium was not consistently evaluated but rather defined based on clinical parameters, such as the absence of episode recurrence for more than a year (Koukopoulos et al. 2013) or a reduction in the frequency of episodes or hospitalizations (Haag et al. 1987). In the present study, for the first time, we have substantiated these observations using a reliable assessment tool, the Alda scale, in conjunction with a multivariate statistical model. Our results align with recent meta-analytic findings (Hui et al. 2019) that aggregated the aforementioned observations, indicating the MDI sequence cycling as a robust predictor of a positive response to lithium, with a low level of heterogeneity across the included studies.

It is interesting to highlight that recent research has largely overlooked the consideration of cycle type in assessing lithium response. This trend could be attributed to the declining use of life charts in systematically evaluating the clinical course of BD, potentially due to the time and effort required for an accurate reconstruction of the clinical trajectory of patients. Our findings underscore the importance of a lifetime approach to the treatment of patients with BD (Sani et al. 2017) and to systematically consider the type of cycle in patients with BD, in order to formulate clinical prognoses and potentially guide more effective and safer treatment strategies.

In terms of temperament traits, our findings revealed that hyperthymic and irritable temperaments are distinct predictors of a favorable response to lithium treatment. The results regarding hyperthymic temperament corroborates a prior study that demonstrated a positive correlation between lithium response and hyperthymic temperament (Rybakowski et al. 2013). Importantly, our findings confirmed this observation within a larger sample size and using a comprehensive multivariate statistical model. Clinically, these results may be indicative of lithium’s enhanced efficacy in the context of acute euphoric mania, a condition often associated with hyperthymic temperament (Perugi et al. 2001). Furthermore, hyperthymic traits tend to be linked with a predominant manic/hypomanic polarity (Azorin et al. 2015) which, in turn, is predictive of a positive lithium response phenotype (Scott et al. 2020).These findings align with the historical notion of lithium’s primary antimanic action, as first observed by Cade (Cade 1949), and are consistent with the association we identified between MDI cycle and lithium response, further supporting the relevance of the manic/hypomanic phase in predicting a favorable response to lithium treatment.

The link between irritable temperament and positive response to lithium may also be due to lithium’s antimanic effects, which counteract the intrinsic excitatory nature of irritable traits. In line, patients with irritable temperament spend more time in manic/hypomanic phases (Miola et al. 2021), are more frequently diagnosed as BD type I (Pompili et al. 2014) and present with more prodromal symptoms prior to the first manic/hypomanic episode (Zeschel et al. 2015).

It is important to acknowledge that our findings regarding irritable temperament do not match the study conducted by Rybakowski et al. (Rybakowski et al. 2013). Furthermore, they appear to contradict well-established observations that suggest a positive association between irritable temperament and mixed states (Tundo et al. 2023), which have in turn been linked to a less favorable response to lithium (Etain et al. 2017; Sportiche et al. 2017). However, it is important to emphasize that these two studies have not been consistently replicated, and recent meta-analytic data suggest that the relationship between mixed states and lithium response is inconclusive and lacks consistency (Hui et al. 2019). Additionally, lithium is increasingly recognized in clinical practice as an effective treatment for managing mixed states (Sani and Fiorillo 2020).

Our study revealed that early adverse events, specifically emotional and physical abuse, substantially predict an inadequate response to lithium treatment in patients with BD. This finding is consistent with a prior study by Etain et al. (2017), which reported an association between suboptimal response to lithium and physical abuse, but not emotional abuse. Nevertheless, the authors cautioned that the absence of association with emotional abuse may be attributed to limited statistical power. Cascino et al. (2021) is the only other study that evaluated patients using both the Alda scale and the CTQ-SF, showing similar results.

Our results show that the deleterious impact of early adverse events on treatment response may be mediated by the absence of a reduction in the overall illness activity. This aligns with the observed worsened clinical course associated with childhood trauma in BD, characterized by a higher frequency of episodes, increased hospitalizations, elevated suicidality and self-harm, compromised psychological functioning, and greater cognitive impairment (Barczyk et al. 2023; Daruy-Filho et al. 2011; Janiri et al. 2023b; Janiri et al. 2023a; Köhler-Forsberg et al. 2024). This may exert a particular influence on the clinical course of patients with an unstable temperament and high nervous reactivity, characteristics that are prevalent among most BD type II patients (Koukopoulos et al. 2006).

The substantial percentage of patients with BD reporting childhood adversities may also be linked to the relatively high prevalence of patients who do not achieve an optimal response to lithium, as reported in other studies using the Alda scale (Sportiche et al. 2017). Childhood trauma may impact various biological pathways, possibly not fully countered by lithium treatment, explaining why some patients on lithium remain at high risk for mood recurrences. Trauma may affect pathways related to inflammation, neuroplasticity, circadian systems, and premature aging (Gill et al. 2020; Janiri et al. 2017; Ridout et al. 2018). It can lead to changes in Brain-derived neurotrophic factor (BDNF) levels and inflammatory markers, potentially creating an imbalance between neuroplasticity and proinflammatory cytokines (Gill et al. 2020; Misiak et al. 2020). Molecular hypotheses related to the circadian system and telomere length are also relevant, with childhood trauma possibly contributing to poor lithium response due to long-lasting biological consequences (Ridout et al. 2018). Additionally, childhood trauma can alter brain structures, such as hippocampus and amygdala (Janiri et al. 2017), which may further impact lithium response (Sani et al. 2018).

Consistently with prior observations (Scott et al. 2017, 2020; Sportiche et al. 2017), although not confirmed by meta-analytic results (Hui et al. 2019), we also noted a link between lithium non-response and lifetime substance use. Additionally, we found a positive association between BD type I diagnosis and early age at onset with favorable lithium response. The first observation on BD subtypes aligns with previous studies (Sportiche et al. 2017) and supports the prominence of lithium’s antimanic effect. The second finding is contentious because it contradicts prior research, which has shown a positive association between lithium response and later age at onset (Hui et al. 2019; Lee et al. 2020). However, it’s worth noting that one previous study found that lithium responders were more likely to have an earlier onset compared to non-responders (Garnham et al. 2007), and another study identified age at onset between 15 and 35 years as a predictor of lithium response (Scott et al. 2020). Nonetheless, none of these associations in our study survived multivariate analyses.

Before presenting our conclusions, we need to acknowledge certain limitations that could affect the generalizability of our findings. Firstly, the cross-sectional design of our study limits our ability to establish causal relationships. However, we mitigated this limitation by employing a multivariate model to predict lithium response, while accounting for relevant confounding factors. Secondly, despite including both BD type I and type II subtypes, there is an imbalance in our sample, with a higher proportion of patients with BD type I (77%) compared to type II (23%), representing a potential limitation in terms of representativeness. Thirdly, clinical variables and treatment response were assessed retrospectively. Nevertheless, we used the Alda scale, which is considered the gold standard for evaluating lithium response (Manchia et al. 2013). Additionally, to reduce the risk of recall bias, we collected information not solely from patient reports but also from family members and close friends (who were present at least at one visit) and reviewed all available medical records. Lastly, while our study features a relatively large sample size, it is still smaller than those in recent genetic and clinical studies (Nunes et al. 2020; Scott et al. 2020). Nonetheless, this study represents the first attempt to simultaneously consider clinical variables, temperament traits psychopathological dimensions, and childhood trauma as potential predictors of lithium response.

In conclusion, our study enhances our capacity to characterize lithium response in individuals with BD by incorporating clinical variables alongside temperament traits, psychopathological traits and early adverse events. Temperament and childhood trauma, when considered alongside the type of cycling, should consistently be factored into the initial phases of BD treatment. The collective evaluation of clinical and psychopathological dimensions may prove to be more predictive of lithium response than the nosological diagnosis alone, as suggested by the current perspective of prominent researchers and clinicians worldwide (McIntyre et al. 2022). Future research endeavors could delve into the biological mechanisms underpinning the associations we have identified and their impact on lithium response.

## Data Availability

The datasets used and/or analysed during the current study are available from the corresponding author on reasonable request.

## Abbreviations

AQ: Aggression Questionnaire
BD: Bipolar Disorder
BDNF: Brain-derived neurotrophic factor
BIS: Barratt Impulsiveness Scale
CI: Confidence Interval
CTQ-SF: Childhood Trauma Questionnaire short form
DSM-5: Diagnostic and Statistical Manual of Mental Disorders 5th edition
MMSE: Mini Mental State Examination
OR: Odds Ratio
SD: Standard Deviation
TEMPS-A-SF: Temperament Evaluation of Memphis, Pisa, Paris, San Diego Autoquestionnaire short form
TS: Total Score
VIF: Variance Inflation Factor
WMA: World Medical Association
