# Correction: Type of cycle, temperament and childhood trauma are associated with lithium response in patients with bipolar disorders

**DOI:** 10.1186/s40345-024-00347-6

**Published:** 2024-07-11

**Authors:** Delfina Janiri, Alessio Simonetti, Mario Luciano, Silvia Montanari, Evelina Bernardi, Giuseppe Carrà, Andrea Fiorillo, Gabriele Sani

**Affiliations:** 1 Department of Psychiatry, Fondazione Policlinico Universitario Agostino Gemelli IRCCS, Rome, Italy; 2 Department of Neurosciences, Section of Psychiatry, Università Cattolica del Sacro Cuore, Rome, Italy; 3 Menninger Department of Psychiatry and Behavioral Sciences, Baylor College of Medicine, Houston, TX USA; 4 Department of Psychiatry, University of Campania “L. Vanvitelli”, Naples, Italy; 5 Department of Medicine and Surgery, University of Milano-Bicocca, Monza, Italy; 6 Division of Psychiatry, University College London, London, UK

**Correction: International Journal of Bipolar Disorders (2024) 12:10** 10.1186/s40345-024-00331-0

In Table 2 of this article (Janiri et al. 2024), the data in the row headed "lithium responders" were mistakenly listed under the row headed "non-responders" and vice versa.

The corrected version of Table 2 is given in this correction.

**Table 2:** Psychopathological dimensions, temperament traits and childhood trauma according to lithium response (n = 201)

	Lithium non-responders (n = 140)	Lithium responders (n = 61)	*F*	df	*P*
*TEMPS-A-SF*					
Cyclothymic	5.08 ± 3.78	5.07 ± 3.20	0.001	1	0.981
Dysthymic	3.35 ± 2.48	2.75 ± 2.16	2.63	1	0.106
Irritable	1.72 ± 1.96	2.80 ± 2.50	10.33	1	0.002**
Hyperthymic	3.59 ± 2.32	4.78 ± 2.17	11.39	1	0.001**
Anxious	1.30 ± 1.21	1.52 ± 1.31	1.27	1	0.260
*CTQ-SF*					
Emotional abuse	9.94 ± 5.30	7.61 ± 3.47	9.87	1	0.002**
Physical abuse	7.74 ± 4.27	5.96 ± 2.19	9.49	1	0.002**
Sexual abuse	6.94 ± 3.64	5.70 ± 1.50	6.49	1	0.012*
Emotional neglect	12.03 ± 5.18	10.49 ± 4.98	3.78	1	0.053
Physical neglect	7.89 ± 3.08	7.06 ± 2.41	3.45	1	0.065
*BIS*					
Attentional	15.84 ± 3.93	15.18 ± 3.58	1.10	1	0.295
Motor	22.09 ± 4.71	21.35 ± 4.12	1.01	1	0.316
Non-planning	26.81 ± 5.05	25.80 ± 5.46	1.45	1	0.230
Total	64.80 ± 10.66	62.33 ± 10.36	2.08	1	0.151
*AQ*					
Physical	17.12 ± 7.04	16.58 ± 5.84	0.235	1	0.628
Verbal	14.70 ± 4.34	13.85 ± 3.49	1.64	1	0.202
Anger	16.78 ± 5.52	16.87 ± 4.96	0.01	1	0.920
Hostility	20.56 ± 6.74	19.59 ± 5.37	0.89	1	0.347
Total	69.05 ± 18.63	67.28 ± 15.23	0.384	1	0.536

*df* degrees of freedom, *P* significance level, *SD* standard deviation, *TEMPS-A-SF* Temperament Evaluation of Memphis, Pisa, Paris and San Diego-Autoquestionnaire, short form, *CTQ-SF* Childhood Trauma Questionnaire short form, *BIS* Barratt Impulsiveness Scale, *AQ* Aggression Questionnaire

*p < 0.05; **p < 0.01; ***p < 0.001

## References

[CR1] Janiri D, Simonetti A, Luciano M, Montanari S, Bernardi E, Carrà G, Fiorillo A, Sani G (2024). Type of cycle, temperament and childhood trauma are associated with lithium response in patients with bipolar disorders. Int J Bipolar Disord..

